# Einstein São Paulo: its metrics and some related issues

**DOI:** 10.1590/S1679-45082016ED3922

**Published:** 2016

**Authors:** Jacyr Pasternak, Sidney Glina

**Affiliations:** 1 Hospital Israelita Albert Einstein, São Paulo, SP, Brazil.

The impact factor (IF) is the main tool to evaluate a journal nowadays. When you suggest a journal to submit a paper, the first question one may consider is “What is its impact factor?”. Everywhere investigators are pushed to publish their articles on high IF journals. It is a perverse situation, because some journals, like Science or Nature, have a rejection rate greater than 95% and only accept the very important articles that will be highly cited, thus contributing to increase their IF. It is much more difficult for journals without an IF, or with a low IF, to get the index or increase it.

Garfield conceived the IF in 1955, when he published an article in Science suggesting how it should be calculated. His idea was discussed and the Science Citation Index was developed and published in 1964.^(1)^ Thomson Reuters bought The Science Citation Index and publishes it today as the Journal Citation Report. The way this metrics is calculated is easy: all citations of all articles published in the past 2 years, or occurring in the coming year, in journals that are part of the Web of Science, are retrieved and divided by all articles published in the journal during those 2 years. This is the IF of the journal. However, getting an IF is not so easy; it does not matter if you are indexed on PubMed, or have published the peer-reviewed texts on time, for years. The journal must to be evaluated by Thomson Reuters reviewers, who have to agree that the publication deserves to be included in their collection. However, the criteria used for selection have not been always clear.

Some points should be clarified before analyzing the IF. It cannot be used to compare disciplines or specialties, since some publish much more than others. The IF is a measure of the quality of the journal, not of its articles. Actually, a very bad paper can originate many citations stating how bad it is.^(2)^ Review articles that usually do not contain original research are far more cited than other articles. The citation index can be manipulated: as early as in 1977, one editor of Leukemia suggested to its authors that he would appreciate if they cited articles in his journal…^(3)^ A more subtle trick occurred in Brazil, when four journals decided to improve their citation numbers by citing each other…^(4)^

The IF should not be used to analyze scientific productivity or payments to scientists, as observed in some countries. Nor should it be utilized to make decisions about grants, contracts or to qualify graduate programs. The IF assessed by Thomson Reuters mirror only articles published in English, and some types of research. Investigations about neglected tropical diseases, for example, are reflected only when the condition affects more civilized places, like Zika virus going to Florida.^(5)^ Again, the IF is a measure of the journal, not of the scientific merit of the article or its authors.

There are other indexes measuring citation numbers besides that by Thomson Reuters, such as the Scimago index. The Brazilian agencies, including the Coordination for the Improvement of Higher Education Personnel (CAPES - *Coordenação de Aperfeiçoamento de Pessoal de Nível Superior*) and the National Council for Scientific and Tecnological Development (CNPq - *Conselho Nacional de Desenvolvimento Científico e Tecnológico*). use the IF to qualify graduate programs; perversely, it results in Brazilian authors and graduate students trying to publish in journals with an IF greater than one. In addition, very few Brazilian journals meet this criterion. The Brazilian scientific organizations have tried to develop good journals, with clear peer review rules to improve the quality of research in the country. This has been more evident in biological and medical journals. The problem is that with those CAPES and CNPq rules, excellent papers are submitted to international journals rather than to our Brazilian publications.

We agree that publishing in English is a must: the articles published in Portuguese are condemned to be unknown by the international scientific community. Einstein (São Paulo) is one of the very few Brazilian journals that translate free of charge all articles accepted for publication that are submitted in Portuguese. We do peer review, do it fairly and within reasonable time − and this topic could render another editorial. We try to have the best articles to publish but, obviously, they can be only as good as the universe they are part of. Moreover, we are sure that excellent articles are submitted to international journals and, when rejected, are sent to us. Actually, some articles are not good enough and we also reject them. Our standards do not differ from other journals, be them Brazilian or international.

Currently, einstein (São Paulo) has a Scimago index and is among the emerging journals registered on the Web of Science: if we are cited enough in the next two years, we will have an official IF from Thomson Reuters. We would like to point out that the IF is only one of the elements that make the reputation of a journal; for sure, it is not the single one and might not be considered the best item.
